# Identification of Mutant Genes and Introgressed Tiger Salamander DNA in the Laboratory Axolotl, *Ambystoma mexicanum*

**DOI:** 10.1038/s41598-017-00059-1

**Published:** 2017-01-31

**Authors:** M. Ryan Woodcock, Jennifer Vaughn-Wolfe, Alexandra Elias, D. Kevin Kump, Katharina Denise Kendall, Nataliya Timoshevskaya, Vladimir Timoshevskiy, Dustin W. Perry, Jeramiah J. Smith, Jessica E. Spiewak, David M. Parichy, S. Randal Voss

**Affiliations:** 1 Department of Biology, University of Kentucky, Lexington, KY 40506 USA; 2 Lafayette High School, Lexington, KY 40503 USA; 3 Transposagen Biopharmaceuticals, 535 W 2nd Suite l0, Lexington, KY 40508 USA; 4 Department of Biology, University of Washington, Seattle, WA 98195 USA; 5 School of Integrative Biology, University of Illinois, Urbana-Champaign, Urbana IL 61801 USA; 6 Department of Biology, University of Virginia, Charlottesville, VA 22903 USA

## Abstract

The molecular genetic toolkit of the Mexican axolotl, a classic model organism, has matured to the point where it is now possible to identify genes for mutant phenotypes. We used a positional cloning–candidate gene approach to identify molecular bases for two historic axolotl pigment phenotypes: white and albino. White (*d*/*d*) mutants have defects in pigment cell morphogenesis and differentiation, whereas albino (*a*/*a*) mutants lack melanin. We identified in white mutants a transcriptional defect in *endothelin 3* (*edn3*), encoding a peptide factor that promotes pigment cell migration and differentiation in other vertebrates. Transgenic restoration of Edn3 expression rescued the homozygous white mutant phenotype. We mapped the albino locus to *tyrosinase* (*tyr*) and identified polymorphisms shared between the albino allele (*tyr* ^*a*^) and *tyr* alleles in a Minnesota population of tiger salamanders from which the albino trait was introgressed. *tyr* ^*a*^ has a 142 bp deletion and similar engineered alleles recapitulated the *albino* phenotype. Finally, we show that historical introgression of *tyr* ^*a*^ significantly altered genomic composition of the laboratory axolotl, yielding a distinct, hybrid strain of ambystomatid salamander. Our results demonstrate the feasibility of identifying genes for traits in the laboratory Mexican axolotl.

## Introduction

The Mexican axolotl (*Ambystoma mexicanum*) is the primary salamander model in biological research. Living stocks established over 150 years ago continue to facilitate research in multiple areas, including development, evolution, and regeneration^1^. In recent years, genomic resources have been developed to enable comparative genomics, quantitative trait locus mapping, gene expression analysis, and the creation of transgenic lines and targeted knock-outs^2–9^. These efforts have brought the axolotl closer to becoming a genetic model organism, that is, a model that can be used to identify genes for phenotypes that are best studied using the axolotl. Yet gene identification remains a formidable challenge. The axolotl’s massive genome (10x the size of the human genome)^9^ and lengthy time to maturity (1–1.5 years) present hurdles for map-based cloning approaches that are typical of genetic model organisms.

Two historic, single-locus, recessive traits that are maintained in domestic axolotl populations present an opportunity to establish map-based cloning in the axolotl, and secondarily, to identify the genetic bases for long-studied phenotypes. The first of these, “white”, was discovered among the original 33 founder axolotls collected from Xochimilco, Mexico in 1863^10^. One male exhibited white coloration, strikingly different from the “dark” mottled green and black of the wild type. The white phenotype results from a recessive allele, *d*, at a single locus^11^ and white mutants figured prominently in demonstrating that vertebrate pigment cells are derived from neural crest cells^12^. Studies over several decades established that white mutants have defects in the morphogenesis of black melanophores and yellow xanthophores^11,13–17^. In wild-type (*D*/-) embryos, melanophores and xanthophores begin to differentiate in the premigratory neural crest along the dorsal neural tube and migrate from this position to cover the flank. In white (*d*/*d*) mutants, fewer melanophores and xanthophores differentiate, and most fail to migrate. Subsequently, melanophores and xanthophores that did differentiate are lost, presumably by cell death, and late-appearing, iridescent iridophores fail to develop. Thus, white larvae and adults are typically devoid of all pigment cells, although occasional adults have been reported to develop patches of melanophores^18^, likely arising from post-embryonic stem cells^19^. Additional analyses have indicated that the white gene acts non-autonomously to pigment cells in promoting their development; for example, wild-type epidermis or subepidermal extracellular matrix can rescue pigment cell migration in white mutants^12,14,17,20^. Despite its striking phenotype, natural origin and historical significance, the genetic basis of the white phenotype has been a mystery^21^.

Another historic axolotl pigment phenotype is albino (*a*), in which melanophores develop but remain unmelanized^22^. Surprisingly, the albino phenotype did not arise within the axolotl lineage but was instead established by interspecific hybridization^23^. In 1962, a terrestrial tiger salamander (*A*. *tigrinum*) lacking melanin was collected near Foot Lake, Willmar Minnesota and after almost a year in captivity, it was gifted to Rufus Humphrey at Indiana University. Humphrey knew it was possible to cross a terrestrial, metamorphic tiger salamander to an aquatic paedomorphic axolotl by *in vitro* fertilization^24^. Unfortunately, embryos from the interspecific cross he performed began to die. As a work around, Humphrey and colleagues used cutting edge technologies for the time – somatic cell nuclear transfer and microsurgery – to create embryos that carried the albino gene. Descendants of these species hybrids were crossed into various axolotl strains and are maintained today in the Ambystoma Genetic Stock Center (AGSC; University of Kentucky).

Here we report the molecular genetic characterization of the first axolotl mutant phenotypes. Genomic locations were established by meiotic mapping of mutant phenotypes to regions harboring candidate genes, *endothelin 3* (*edn3*) for white and *tyrosinase* (*tyr*) for albino. Confirming identification of the white gene, we found a defect in *edn3* ^*d*^ expression in white mutants, performed a knockdown of *edn3* in wild-type to phenocopy white, and rescued the mutant via transgenic restoration of Edn3 expression. For albino, we identified the causative lesion in *tyr* ^*a*^ and used genome editing to delete *tyr* coding sequence in wild type, recapitulating the albino phenotype. Surprisingly, we also found through pedigree analysis that all individuals of the current AGSC population are descendants of the albino tiger salamander and 88% of current adult wild-type axolotls carry *tyr* ^*a*^. Moreover, the majority of *tyr* ^*a*^ alleles are associated with a larger than expected segment of the ancestral haplotype, and all individuals retain a small contribution from the tiger salamander genome. Our study illustrates the feasibility of using classic and cutting-edge genetic and genomic tools to target historically significant traits, a prelude to investigating additional traits (e.g., paedomorphosis and regeneration) that are best studied in the axolotl.

## Results

### Axolotl white is associated with the Edn3 locus

**Figure 1:**
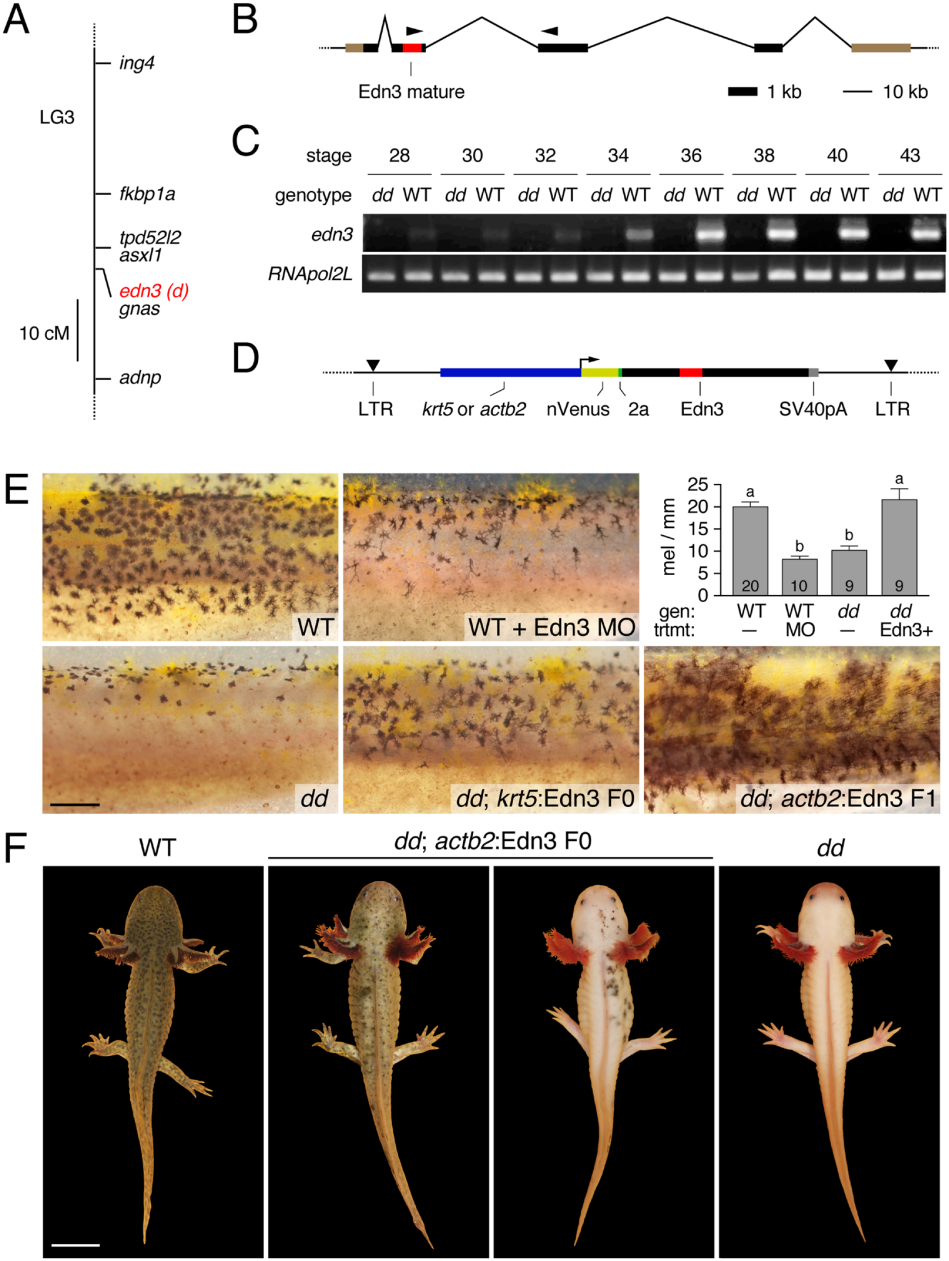
White locus corresponds to *edn3*. (**A**) Mapping of white mutant phenotype (*d*) to *edn3*. cM, centimorgan. (**B**) Genomic structure of axolotl *edn3* showing exons (thick lines) and introns (thin lines). Red, encoding mature Edn3 peptide. Brown, untranslated regions. Arrowheads, primers used for RT-PCR. Note scales differ for exons and introns. (**C**) Expression of Edn3-peptide encoding transcript in wild type (WT) but not white mutant (*dd*) embryos. See Supplementary Fig. 9 for uncropped gel images. (**D**) Construct design for transgenic restoration of Edn3 expression (see text). (**E**) Phenotypes resulting from *edn3* morpholino knockdown in WT (Edn3 MO; upper) and transgenic rescue of mutant (*dd*) with *krt5*:Edn3 or *actb2*:Edn3 transgenes in F0 or F1 generations, respectively. Upper right plot shows mean ± SE total numbers of melanophores per mm (see Methods) across genotypes (“gen”) and treatments (“trtmt”). In comparison to unmanipulated WT larvae (“WT, −”), morpholino knockdown of Edn3 (“WT, MO”) resulted in significantly fewer melanophores, indistinguishable in number from those of unmanipulated mutants (“*dd*, −”). By contrast, injection of dd mutants with krt:Edn3 transgene to express Edn3 in epidermis (“*dd*, Edn3+”). Letters above bars indicate groups not significantly different in post hoc comparisons of means (overall ANOVA: *F*_3,44_ = 22.3, *P* < 0.0001). Numbers within bars indicate numbers of larvae examined. (**F**) Adult WT (left), white mutant (right) and transgenics expressing Edn3 mosaically, illustrating range of phenotypes observed. Scale bars: 0.5 mm in E; 2 cm in D. Mathew Niemiller is credited with taking the photographs in Fig. 1F.

Previously, the white gene was mapped near anonymous EST markers on linkage group 3 of the *Ambystoma* meiotic map^25^. One of these markers exhibited significant sequence identity to *asxl1*, which in chicken is located on chromosome 20 (NCBI Gene ID: 428158; 20:10,359,763 bp). An orthologue of the linked gene *fkbp1a* (NCBI Gene ID: ID: 374233; 20:10,023,227 bp) was then mapped 9 cM from *asxl1* in *Ambystoma* ^26^, thus identifying a conserved chromosomal segment adjacent to the *white* locus (Fig. 1A). Making the assumption that in this region, gene orders are highly similar in the chicken and the salamander genome^3^, we assessed genes in the vicinity of *asxl1* and *fxbp1a* as candidates for white. One gene – *edn3* (NCBI Gene ID: 768509; 20:11,001,554 bp) – received top priority because of its non-autonomous functions in melanoblast migration and proliferation, similar to that inferred for white^14,17,27–29^. A Sal-Site (RRID:SCR_002850)^30^ EST contig (V4 contig436215) containing partial *edn3* sequence was used to identify single nucleotide polymorphisms for a unique allele (*edn3* ^*d*^), that was always homozygous in all *d*/*d* individuals (N = 40), but absent or heterozygous in all wild-type individuals (N = 13).

### Correspondence of white (*d*) and *edn3*

We cloned full-length *edn3* transcripts from hatching stage wild-type individuals (stage 41)^31^ and aligned the sequences to a genomic contig (NCBI Accession Pending) that was assembled using BACs and DNA sequence data from an initial axolotl genome assembly^9^. Axolotl *edn3* is structurally similar to other vertebrate orthologues, with four exons of coding sequence; exon 2 is predicted to encode the 21 amino acid mature peptide that functions as a ligand for Endothelin receptor type B (Fig. 1B). Sequencing of wild-type and white cDNAs from embryos revealed splice variants in each background yet white cDNAs consistently lacked exon 2. RT-PCR using primers to detect coding sequence for the mature Edn3 peptide revealed initially low but increasing levels of transcript in wild-type embryos beginning during stages of neural crest migration, but no expression in white mutants (Fig. 1B,C).

Mammalian and avian mutants for Edn3 signaling have defects in pigmentation^28,32–34^, yet mutants for Edn3 and Endothelin receptor B in zebrafish have normal early larval pigment patterns^35,36^. We predicted that if *d* corresponds to *edn3*, then knockdown of Edn3 should phenocopy white, more similar to mammals than to zebrafish. We found that injection into wild-type of a translation-blocking morpholino targeted to *edn3* resulted in fewer melanophores over the lateral flank as compared to controls (Fig. 1E, top panels).

Finally, we predicted that restoring *edn3* expression to white mutants should rescue wild-type pigmentation. Accordingly, we constructed *Tol2* transgenes encoding axolotl Edn3 linked by peptide-breaking 2a sequence to nuclear-localizing Venus fluorophore. To drive Edn3 we used regulatory elements from zebrafish for *krt5* (2.9 kb; skin) or *actb2* (5.3 kb; ubiquitous) (Fig. 1D; Supplementary Fig. S1). Consistent with prior inferences that white acts in the epidermis to influence pigment cell morphogenesis, injection of white mutant embryos with *krt5*:Edn3 transgene rescued melanophore numbers and distributions to phenotypes indistinguishable from wild-type (Fig. 1E upper right and lower panels; Supplementary Fig. S2A) and also rescued xanthophore migration (Supplementary Fig. S2B). Ubiquitous expression of Edn3 in white mutants by injection (F0) or germline transmission (F1) of *actb2*:Edn3 likewise rescued melanophores and xanthophores, though high cell densities precluded identifying individual cells for quantification (e.g., Fig. 1E, lower right). At later stages, larvae injected with *krt5*:edn3 transgene exhibited only minimal rescue phenotypes (Supplementary Fig. S2C); indeed, *krt5*:Edn3 expression could not be detected post-embryonically (not shown), presumably because transgenic cells in the mosaic yet proliferative epidermal environment had been depleted, or because the transgene itself had been silenced. By contrast, embryos injected with *actb2*:Edn3 (F0) exhibited moderate to substantial rescue phenotypes in the adult (Fig. 1F; individuals expressing *actb2*:Edn3 as F1s were not viable post-embryonically so their phenotypes could not be assessed). Together, genetic mapping, expression analyses, morpholino phenocopy and transgenic rescue all strongly support the inference that the white gene, *d*, corresponds to *edn3*.

### Axolotl albino results from a tiger salamander-derived *tyr* allele

**Figure 2:**
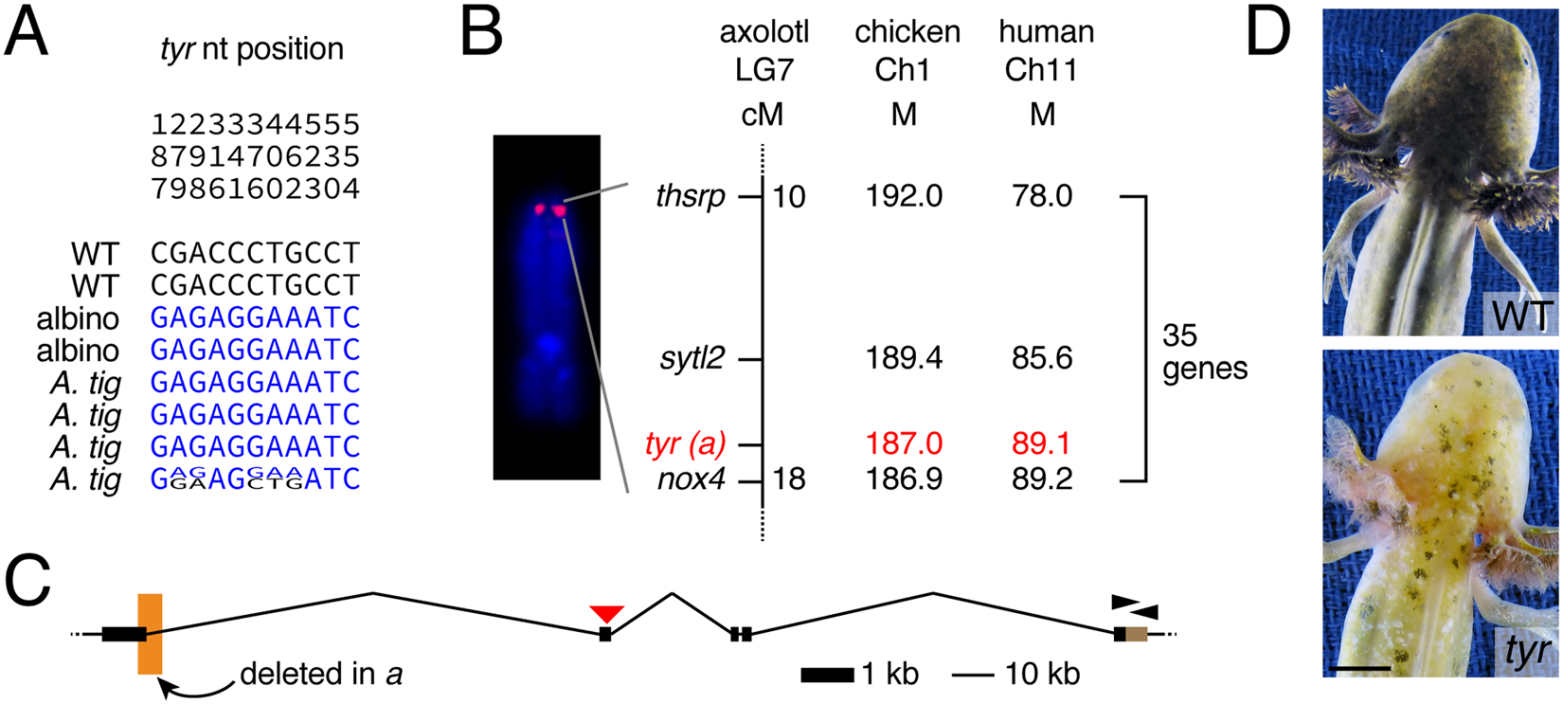
Albino results from a mutation in tiger salamander *tyr*. (**A**) SNPs identified in the *tyr* 3′ UTR of WT axolotls, albino axolotls, and wild caught tiger salamanders. (**B**) FISH and genetic mapping localizes *tyr* to the distal tip of linkage group 7. M, megabases. (**C**) Genomic structure of *tyr*. Orange box, region deleted in *tyr* ^*a*^. Black arrowheads, primer sites for identification of SNPs. Red arrowhead, site targeted for TALEN mutagenesis. (**D**) WT and TALEN-targeted *tyr* mosaic larva, phenocopying the albino mutant. Scale bar: 5 mm.

Similar to white, we reasoned that *albino* might map to a locus associated with albinism in other amphibians^37,38^, including axolotl^39^, and so focused our efforts on *tyr* and *oca2*. *tyr* encodes an enzyme that functions in melanin synthesis while *oca2* encodes an integral membrane protein that functions to maintain melanosomal pH^40,41^. We isolated DNA from adult albino (*a*/*a*) and wild-type axolotls and amplified fragments from the 3′ UTRs of *tyr* and *oca2*. DNA sequencing revealed 11 SNPs within a 459 base pair (bp) region of *tyr* that differed between albino and wild-type axolotls (Supplementary Fig. S3); no diagnostic SNPs were discovered for *oca2*. The relatively large number of SNPs identified for *tyr* was striking because polymorphisms occur at a lower frequency in AGSC axolotls (see below). This observation is consistent with derivation of the *tyr* allele (*tyr* ^*a*^) from tiger salamander. We obtained *tyr* DNA sequences from tiger salamanders collected near the Minnesota site where the albino tiger salamander was discovered in 1962: three yielded sequences identical to *tyr* ^*a*^ and the fourth yielded alleles more similar to *tyr* ^*a*^ than to *tyr* of wild-type axolotl (Fig. 2A). Fluorescence *in situ* hybridization (FISH) placed *tyr* near the tip of a chromosome that corresponds to linkage group 7 in the *Ambystoma* genetic map (Fig. 2B). Sequencing of additional axolotls revealed that all albino individuals were homozygous for tiger salamander *tyr* ^*a*^ (N = 12) whereas wild-type individuals were either heterozygous for *tyr* ^*a*^ or homozygous for axolotl-derived *tyr* alleles (N = 11).

To search for causative mutations, we screened a BAC library and identified two overlapping BAC clones (AMMCBa_160B14, AMMCBa_31L9; GenBank Locus KU684456) that contained the entire *tyr* coding sequence, spanning 219,419 bp of genomic DNA, more than 100 kb longer than other vertebrate *tyr* orthologs. Alignment of wild-type and albino cDNAs to the genomic sequence revealed a 142 bp deletion of *tyr* ^*a*^ coding sequence at the end of exon 1 (extending ~3 kb in to intron 1) that results in a frame-shift and premature stop codon (Fig. 2C). Confirming the requirement for this locus in melanin synthesis, targeted mutagenesis of *tyr* using a TALEN phenocopied albino (Fig. 2D; Supplementary Fig. S4)^39^.

### Establishment of a distinct, hybrid laboratory strain of ambystomatid salamander

**Figure 3:**
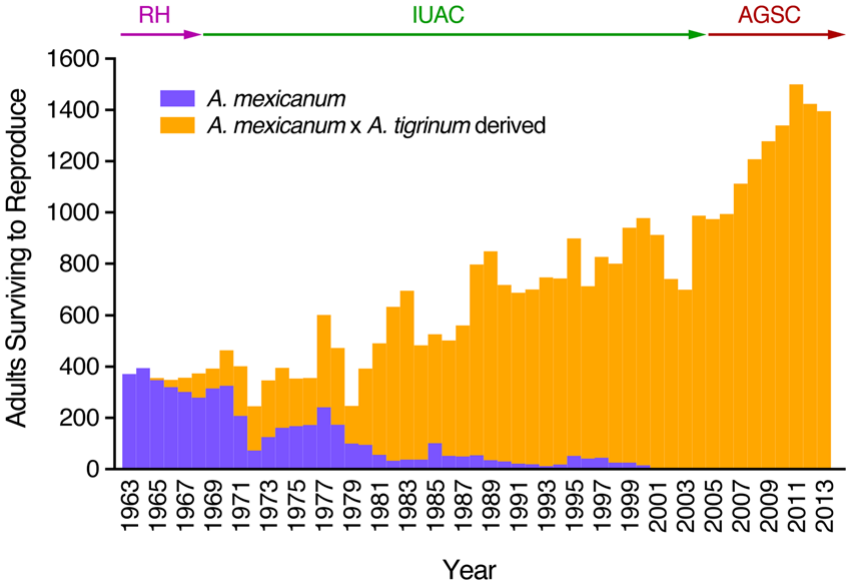
Census of hybrid and historical adult axolotls surviving to reproduce (N = 21,938) from 1963–2013. Historical axolotls (blue) declined in favor of axolotls derived from the ancestral hybridization with albino tiger salamander (orange). RH, Rupert Humphrey (Indiana University); IUAC, Indiana University Axolotl Colony; AGSC, Ambystoma Genetic Stock Center (U. Kentucky).

To learn the fate of introgressed tiger salamander DNA in the laboratory axolotl population we compiled historical studbook data. From 1964 through 2013, axolotl–tiger salamander hybrid descendants were mated 29,945 times and 4,884 of these spawns yielded descendants in the current AGSC population. Only 4% of these retained spawns (N = 196) were backcrosses of hybrids to axolotls. In theory, this number of backcrosses would effectively remove tiger salamander DNA after the initial axolotl x tiger salamander cross, if performed recurrently within a single founder lineage. Yet, the backcrosses were distributed instead across three different founder lineages (Supplementary Fig. S5). Over time, hybrid descendants were crossed to all existing axolotl strains, and by 2000, all axolotls in the collection traced their ancestry to the 1963 axolotl x tiger salamander hybridization cross (Fig. 3). Pedigree analysis^42^ predicts that an average AGSC axolotl genome contains 5.8 ± 1.0% tiger salamander DNA.

To test directly how much tiger salamander DNA is still associated with *tyr* ^*a*^ specifically, we compared 3′ UTRs for *nox4*, *stl2*, and *thrsp* among 2 wild-type axolotls, 2 albino axolotls, and four tiger salamanders. Albino axolotls and tiger salamanders shared haplotypes at all three loci that were absent from wild-type axolotls (Supplementary Fig. S3), indicating ≥8 cM of contiguous genomic sequence predicted by synteny comparisons to include orthologs of 30 human protein-coding genes (Supplementary Table S6). Examining 48 additional *tyr* ^*a*^ chromosomes in albino mutants, we found evidence of historical recombination between *thrsp* and *tyr* in only 12 cases. Assuming absence of double crossover events within this short region, the majority (35/48, 73%) of *tyr* ^*a*^ alleles are associated with ≥7 cM of tiger salamander genomic DNA. Computer simulations assuming random mating, no selection and recombination as a Poisson process predicted only 11–17% of chromosome segments ≥7 cM, significantly less than the 73% observed (*P* < 0.0001). Thus, longer than expected segments of tiger salamander DNA are retained in linkage with *tyr* ^*a*^ in the laboratory axolotl.

We next tested for tiger salamander DNA distributed throughout the genome by comparing 158 orthologous axolotl and tiger salamander ESTs chosen from across the Ambystoma genetic linkage map. Conservatively, we sampled DNA sequences from 12 axolotls predicted^42^ to carry the largest proportion of an axolotl founder genome. Overall, we identified tiger salamander alleles at 26 loci; one locus was fixed (24/24: E45A6) and two loci nearly fixed (22/24: E35G8, E50G6) for tiger salamander alleles (Supplementary Table S7). On average, each axolotl carried tiger salamander DNA at 12 of the 158 EST markers, suggesting that at least 7.6% of genes in the axolotl genome contain tiger salamander DNA.

## Discussion

In this study we identified genes for the first axolotl color phenotypes – white and albino – two of the most famous of all axolotl traits. A single, white axolotl was collected by a French military expedition along with 33 wild-type axolotls from Xochimilco, Mexico in 1863^10^. These salamanders survived the voyage across the Atlantic Ocean and Auguste Duméril established a thriving population at the Muséum National d’Histoire Naturelle in Paris. Over decades, axolotls were distributed across Europe to researchers and aquarists, and then in 1932 they were shipped back across the Atlantic Ocean to Ross Harrison’s lab at Yale University, and then subsequently to Humphrey at University of Buffalo^43^. The white axolotl received considerable attention from classical embryologists and modern developmental biologists because the phenotype is associated with the morphogenetic behavior of neural crest-derived pigment cells. Several studies have suggested that the white gene product is required non-autonomously by pigment cells during their migration from the neural crest and some have inferred defects in the extracellular matrix of white mutants through which these cells travel^12–17,20,21,44,45^. Our findings that strongly suggest white results from a mutation in the gene encoding secreted Edn3 are consistent with earlier work, and will provide a critical resource for understanding the evolution of endothelin signaling, required for melanocytes in birds and mammals, iridophores in zebrafish, but melanophores, xanthophores and iridophores in axolotls^19,28,32–36,46,47^.

The purity of laboratory axolotl collections was in doubt before the introgression of *A*. *tigrinum tyr* ^*a*^ into the AGSC population. The axolotl is a member of the Tiger salamander complex, a widely distributed group of salamanders in North America that show considerable life history and phenotypic variation in pigment pattern^48^. Reproductive barriers to hybridization have not evolved among closely related, sympatric species or surprisingly, geographically isolated and phylogenetically distinct species like *A*. *mexicanum* and *A*. *tigrinum* ^24^. Brandon^49^ noted that *A*. *mexicanum* and a close tiger salamander relative (*A*. *velasci*) co-occur in the vicinity of Xochimilco, the later exhibiting slight morphological differences and a higher propensity for metamorphosis under laboratory conditions. Smith^10^ speculated that *A*. *velasci* may have been present in the original axolotl collection of 1863, and if not, was almost certainly introduced subsequently into domestic stocks because *A*. *mexicanum* and *A*. *velasci* were typically sold together at Xochimilco markets^49^. During the 1900s, the AGSC population saw multiple introductions of outside “axolotls” to uncover new recessive mutant alleles for developmental research^50^. Similarly, Humphrey^23^ saw the albino tiger salamander as an opportunity to derive a useful new pigment mutant for the axolotl model. It is not known if laboratory axolotl collections exist that approximate the species integrity of Xochimilco axolotls, but the study of such lineages, or derivation of new laboratory lines from the threatened Xochimilco population, would be useful for understanding mechanisms of domestication and hybrid species origins.

It is typical after gene introgression to perform backcrosses for multiple generations to remove donor DNA sequences from the recurrent parent genome^51^. Our review of the AGSC pedigree showed that relatively few backcrosses were performed and after generations of hybrid–hybrid mating, the number of *A*. *tigrinum* hybrid axolotls increased and historical laboratory axolotl lines declined. It is not clear why the historical axolotl lines were lost by 2000. We note that around this time, the natural Xochimilco axolotl population declined precipitously making it difficult if not impossible to introduce new material into the collection^26^. We speculate that *A*. *tigrinum* hybrid salamanders also may have proven to be more viable and fertile for stock production. Indeed, studies of naturally occurring hybrid ambystomatid salamanders suggest they are often more vigorous than the parental species from which they derive^52^.

The majority of albinos in the current AGSC population retain >7 cM of tiger salamander DNA in linkage with *tyr* ^*a*^. The persistence of linked tiger salamander DNA reflects several factors including the recombination frequency for the *tyr* genomic region and the possibility that *tyr* ^*a*^-linked loci may yield selectively advantageous phenotypes. Two lines of evidence support a role for selection. First, the majority of non-albino adult axolotls in the contemporary AGSC population carry *tyr* ^*a*^, suggesting a fitness advantage for heterozygous carriers that historically could not be differentiated from wild-type homozygotes, perhaps owing to the utility of albino embryos for research itself. Second, computer simulations of recombination since the axolotl-tiger salamander hybridization event predicted significantly fewer long chromosome segments than we observed. In the future it will be possible to investigate the selection hypothesis further by comparing fitness traits between *tyr* ^*a*^ individuals and individuals deriving from the TALEN axolotl lines created in this study.

In closing, we note that domestic AGSC axolotls are genetically distinct from the natural, remnant population at Xochimilico^50^. AGSC axolotls should be considered a distinct, hybrid axolotl strain. If an average AGSC axolotl has tiger salamander DNA at 7.6% of loci, then >1000 genes in the genome are expected to be segregating tiger salamander DNA, with implications for design of molecular probes and primers, gene targeting vectors and other approaches. These findings are somewhat reminiscent of zebrafish, in which startlingly high levels of polymorphism are observed between and even within laboratory strains, but also offer opportunities to study genome evolution and allelic effects^53^. Finally, axolotl mutants and naturally occurring phenotypes segregate in hybrid backgrounds according to Mendelian predictions and so the presence of tiger salamander DNA will not hinder efforts to clone other historic axolotl mutants including *eyeless*, *cardiac*, *short*-*toes*, *axanthic*, and *melanoid*, or QTL for developmental traits such as paedomorphosis^4,54,55^ that are best studied in the axolotl.

## Methods

### Animals

All axolotls and tissues in this study were obtained from the Ambystoma Genetic Stock Center (RRID:SCR_006372). Ethical animal procedures performed in this study were approved by the University of Kentucky IACUC committee (protocol 00907L2005) and all methods were carried out in accordance with relevant guidelines and regulations.

### Nucleic Acid Isolation, cDNA synthesis, DNA sequencing, and SNP analysis

DNA was isolated from tail tips by treatment with SDS, RNAse and proteinase K, followed by phenol-chloroform extraction^56^. Total RNA was isolated from whole embryos with Trizol Reagent (Invitrogen) and further purified with RNeasy mini columns and RNase-Free DNase Sets (Qiagen). DNA and RNA quality and quantity were assessed using a Nanodrop ND-1000 spectrophotometer and an Agilent Bioanalyzer, respectively. An iScript cDNA Synthesis Kit (Bio-Rad), with 250–500 ng of axolotl RNA and Oligo dT-priming, was used to synthesize cDNA for *edn3* sequence comparisons and for RT-PCR analysis of *edn3* transcripts. RT-PCR products were generated from two rounds of PCR. The first PCR reaction used 2.5 ul iScript cDNA in a 25 ul reaction with a final concentration of 0.25 mM dNTPs, 1 mM MgCl2, 0.2 nM primer, and 1x Taq Polymerase. The cycling conditions were 1 cycle 94 °C for 30 s, followed by 36 cycles of 94 °C for 30 s, 55 °C for 30 s, 72° for 45 s, and a final extension cycle of 7 minutes at 72 °C. A 2.5 μl aliquot of the first PCR was then used as template for a second PCR. The conditions of the second PCR were identical to the first excepting a higher annealing temperature (60 °C). RT-PCR products were verified by DNA sequencing and resolved on 1% TAE agarose gels. 5′ RACE (Invitrogen) was used to synthesize cDNAs from wildtype and albino axolotls using 250 ng of total RNA and gene-specific primers. The resulting fragments were gel-excised and purified (Qiagen gel extraction kit), TA-cloned into pGEM (Promega), transformed into TOP10 cells (ThermoFisher), and spread on LB-Amp-XGal-IPTG plates. Single colonies were diluted into 70 μl of water and PCR amplified using 2.0 μl of template, 2.5 μl 10x PCR reaction buffer, 2.5 μl of 1.25 mM dNTPs, 1.2 μl of 1.5 mM MgCl, 1.0 ul of 10 μM mix of M13F/R primers, 15.4 μl H_2_O, and 0.4 μl 5x Taq Polymerase. The PCR amplifying conditions were 1 cycle 94 °C for 3 m, 34 cycles of 94 °C for 45 s, 55 °C for 45 s, 72 °C for 30 s, and 1 cycle 72 °C for 7:00 min. DNA sequencing was performed by the ATGC Sequencing Core and Genomics Core Laboratory at the University of Kentucky. SNP genotyping was performed by direct sequencing of purified PCR products and DNA sequencing, or by TaqMan analysis^57^. The primers used for these analyses are listed in Supplementary File S8.

### BAC library screening, isolation, DNA sequencing, and assembly

BAC clones were identified by PCR from a library prepared by the Clemson University Genomics Institute. Briefly, clones from 12 microtiter plates (386 well) were pooled to form superpools, DNA was isolated for each superpool using the PureLink HI Pure Plasmid Maxiprep Kit (Invitrogen), and each superpool was screened by PCR using primers designed to amplify fragments from *edn3* and *tyr* DNA sequences (S7), using PCR conditions described above for amplifying bacterial colonies. The location of positive clones within microtiter plates of a superpool was determined by sequential PCR of plate, column, and row BAC pools. BAC DNA for positive PCR reactions, as visualized on agarose gels, was isolated using the PureLink HI Pure Plasmid Maxiprep Kit (Invitrogen) and then sequenced to 30x depth on a PacBio RS II by the Duke Center for Genomic and Computational Biology. Resulting reads were assembled using the SMRT Analyses 2.1 HGAP pipeline^58^.

#### DNA probe preparation and fluorescence *in situ* hybridization (FISH)

Chromosome preparations at early and mid-metaphase were made from axolotl embryos at neurula stage as described previously^9^. DNA was isolated from *tyr* BAC clones (AMMCBa_160B14, AMMCBa_31L9) using a Qiagen Large Construct kit. BAC-DNA was prepared for *in situ* hybridization by nick-translation using direct fluorophores Cyanine 3-dUTP (Enzo Life Sciences, ENZ-42501) or Fluorescein-12-dUTP (Thermo Scientific, R0101)^59^. BAC-DNA was isolated using Qiagen Large Construct kit (Qiagen Science, 12462). Hybridization of BAC clones was performed^60^ with suppression of unspecific binding, by pre-hybridizing the probe with an unlabeled Cot2 fraction of repetitive axolotl DNA^61^.

### TALEN and Morpholino analyses

TALEN constructs for *tyr* exon 2 genome editing were designed by Transposagen Biopharmaceuticals. The left TALEN binding site is 5-TACAACAATCTCCAGAT-3 and the right TALEN binding site is 5-ATGCCACAGACGAAGGGCCCATA-3. The *edn3* translation blocking morpholino (5-taacagtaaacgcagctccatgaac-3) and missense control morpholino (5-taaaagtaaaagcacctacatcaac-3) were designed by Gene Tools, LLC.

### Embryo microinjection

The embryos for the e*dn3* rescue experiment were generated by crossing axolotls known to be homozygous for wild-type alleles (RRID:AGSC_100A). The embryos for the *tyr* TALEN editing experiment were generated by crossing homozygous wild type to white axolotls (RRID:AGSC_101A). Embryos were collected from females as they were laid. The egg jelly and membrane were manually removed from 1-cell stage embryos using forceps and the embryos were maintained in 40% Holtfreter’s solution with 1x Penicillin Streptomycin, and then transferred into Solution I with 15% Ficoll 400 prior to performing microinjections. A Harvard Apparatus picoinjector was used for microinjections and the pressure was set to 20 PSI with a duration time (30–200 milliseconds) set to deliver 4 nanoliters. For Tol2 injections, 50 pg of Tol2 vector (DNA) and 25 pg of Tol2 transposase (RNA) was delivered per embryo. For TALENS, 400 pg mRNA for each arm of TALEN-*tyr* Ex2 was injected. For edn3 morpholino experiments, 5 pg was injected. After injection, embryos were allowed to recover for 1–4 hours prior to being transferred into 20% Holtfreter’s solution with 1x Penicillin Streptomycin and 5% Ficoll 400. After approximately 18–24 hours, embryos were transferred into fresh 20% modified Holtfreter’s solution, which was changed daily until embryos developed into feeding larvae. Larvae were maintained in 40% Holtfreter’s solution and fed brine shrimp initially, and then after reaching 3 cM, they were fed California blackworms.

### Quantification of melanophores

Melanophore numbers and pattern were assessed in hatchling larvae by determining the position of each melanophore relative to dorsal and ventral margins of the flank. A unit value of 1 was defined as the distance between dorsal margin of the myotome and the boundary between the ventral myotome and yolk mass such that cells falling within this region received values between 0 (dorsal-most) and 1 (ventral); cells that had migrated ventral to the myotomes and over the yolk mass therefore received values between 1 and 2. For analyses of total melanophore numbers, counts were divided by the anterior–posterior distance of the region examined in each larva, yielding linear densities of melanophores per mm and values were *ln*-transformed to correct for heteroscedasticity of residuals prior to analyses^35,62^.

### Pedigree Analyses

The *Ambystoma* Genetic Stock Center (AGSC) has breeding records dating back to 1935. Records for adult animals at one year of age or older (N = 22,927 individuals) were transferred from a Microsoft Access database to form a studbook in the form of a POPLINK database^63^. During this transfer process, records were checked for internal consistency, errors were corrected, and missing data fields populated according to specifications of the POPLINK Version 2.3 User’s Manual. The full AGSC pedigree was pruned to include only the hybrid offspring of the albino tiger salamander (N = 16,542 individuals) using Pedigraph^64^, a software tool for graphing and analyzing large complex pedigrees. This hybrid descendant pedigree was further pruned using lineage pedigree visualization and analysis software^65^ to include only animals that survived to reproduce in the AGSC (N = 2,795 individuals) The resultant albino family breeder pedigree was manually rendered for ten generations (N = 48 individuals) using CmapTools software^66^.

### Length of *A*. *tigrinum* Ancestral Chromosome Segments in Contemporary Axolotl Albino Genomes

AdmixSim^67^ was used to determine the expected frequency distribution of *A*. *tigrinum* chromosome segments within contemporary AGSC axolotl genomes. AdmixSim was developed to simulate admixture in a standard Wright Fisher population with no selection and random mating, where recombination along the length of a single chromosome is modeled as a Poisson process with a rate equal to one Morgan. We simulated the initial 1962 population as containing one axolotl and one tiger salamander chromosome (232 cM – the size of LG7) that were recombined to generate an F1 population of 300 chromosomes, which approximates the average axolotl clutch size. In generation 2, chromosomes were drawn randomly from the admixed population and recombined, and then in generations 3, 4, and 5 backcrosses were simulated by allowing the introduction of tiger salamander chromosomes at frequency 0.5. After generation 5 and through generation 30, 300 chromosomes were sampled each generation and recombined to form the next generation. This scenario models albino FL1 (S5), a lineage that is predicted to yield the least amount of tiger salamander DNA in the current AGSC population. To contrast this scenario, we modeled the scenario for FL4 (S5), where only a single backcross was performed in generation 4. In the 30^th^ generation, 600 axolotl and tiger salamander ancestral chromosome segments were sampled from each of 10 different simulations to generate a frequency distribution. The frequency of chromosome segments exceeding 7 cM was determined for each scenario and compared, using a Chi-square test, to the frequency of 7 cM segments observed among 48 chromosomes sampled from albino axolotls.

### Survey of Dispersed Tiger Salamander DNA in the Axolotl Genome

To test for genome-wide introgression of genetic material from *A*. *tigrinum*, we amplified fragments of axolotl and tiger salamander DNA by PCR for 158 molecular markers from the Ambystoma meiotic map^25^. Axolotl DNA was isolated from 12 representative individuals in the AGSC that were predicted by PMX^42^ to carry the largest proportion of an original axolotl founder genome. DNA was isolated from cryopreserved tissues of two *A*. *tigrinum* collected from Foot Lake, Willmar, Minnesota by Brad Shaffer. The PCR primer sequences are listed in Supplementary File S7. PCR was performed as described above with the following modifications: 150 ng of template was used along with 50 ng of each primer, and the annealing temperature for PCR was either 55 or 60C. PCR products were purified using the E.Z.N.A Cycle Pure Kit (Omega Bio-Tek) and sequenced as described above. Sequences for each marker were compiled into contigs and manually edited using SeqManPro version 8.0.2. To aid in the detection of unique tiger salamander alleles and exclude ancestral polymorphisms, all sequences were aligned by BLASTn to axolotl and tiger salamander EST contigs obtained from Sal-Site (http://www.ambystoma.org/genome-resources/5-gene-and-est-database), and also to transcripts for a close relative of the axolotl (*A*. *andersoni*). Unique tiger salamander haplotypes were identified by manually examining alignments using SeqManPro version 8.0.2. Only those unique tiger salamander alleles found to be 100% identical to an axolotl allele, and not identical to an *A*. *andersoni* allele, were regarded as evidence of tiger salamander introgression.

## Electronic supplementary material

Supplementary Information

Supplementary Table S7
