# Supplementary material for: Identification of Mutant Genes and Introgressed Tiger Salamander DNA in the Laboratory Axolotl, Ambystoma mexicanum
Source: Sci Rep. 2017 Jan 31;7:6. doi: 10.1038/s41598-017-00059-1 (PMC5428337; doi:10.1038/s41598-017-00059-1)
Supplement: Supplementary file 1 — Supplementary Information [file 41598_2017_59_MOESM1_ESM.pdf]

Supplementary Information

Identification of Mutant Genes and Introgressed Tiger Salamander DNA in the Laboratory Axolotl, *Ambystoma mexicanum*

M. Ryan Woodcock, Jennifer Vaughn-Wolfe, Alexandra Elias, D. Kevin Kump, Katharina Denise Kendall, Nataliya Timoshevskaya, Vladimir Timoshevskiy, Dustin W. Perry, Jeramiah J. Smith, Jessica E. Spiewak, David M. Parichy, S. Randal Voss

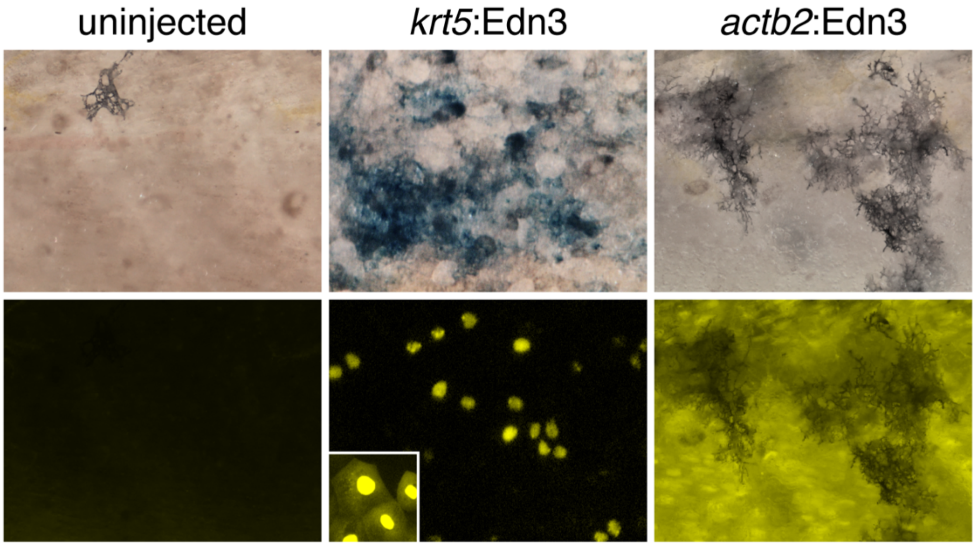

Supplementary Figure S1. Expression of *krt5* and *actb2* driven transgenes in F0 mosaic embryos. Upper, brightfield. Lower, nuclear Venus. Inset shows polygonal shape of epidermal Venus+ cells.

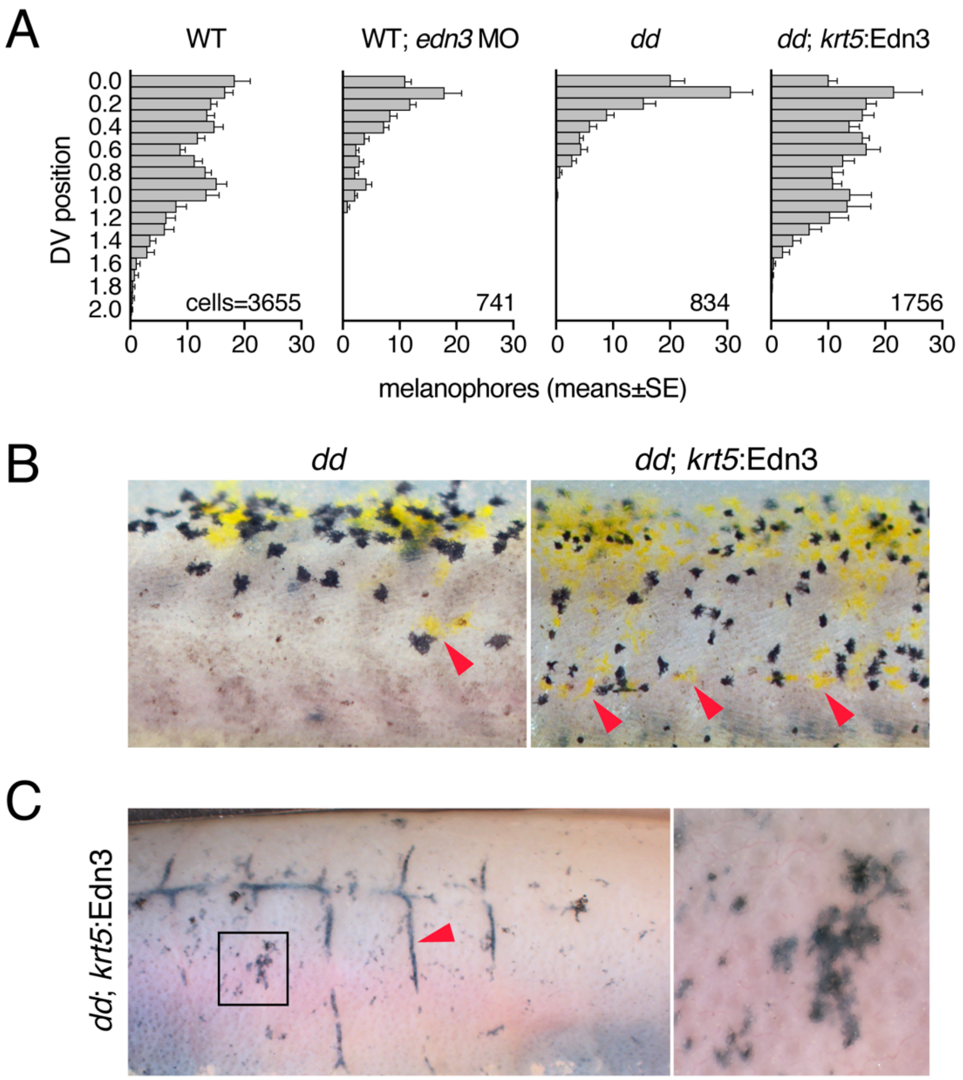

Supplementary Figure S2. Modulation of Edn3 expression in wild-type and white mutant axolotls. rescue of white phenotype by *krt5*:Edn3 expression. (A) In white mutants, *krt5*:Edn3 expression results in more ventral melanophores and xanthophores (arrowheads) as compared to non-transgenic control. (A) Mean±SE numbers of melanophores at different dorsal (0.0) to ventral (2.0) positions on the flank of hatchling larvae (corresponding to individuals in Fig. 1E). Morpholino knockdown of Edn3 in WT embryos (WT; *edn3* MO) results in melanophores failing to reach ventral regions of the flank, as in white mutants (*dd*). By contrast, expression of of Edn3 in skin of white mutants (*dd*; *krt5*:Edn3) restores ventral melanophores, similar to WT. (B) In white mutants, *krt5*:Edn3 expression also restores ventral xanthophores (arrowheads) as compared to non-transgenic control. (C) At later larval stages (~2 months post-fertilization, 6 cm total length), *krt5*:Edn3-injected individuals exhibited melanophores along vertical and horizontal myosepta (e.g., arrowhead) and sparsely across the skin, in contrast to the complete absence of such cells at corresponding stages of non-transgenic white mutants. Boxed region in left panel shown at higher magnification in right panel.

nox4 Nucleotide Position

	501	522-538	547	653
CrA2-wildtype	T	ins	A	T
CrA6-wildtype	T	ins	A	T
CrA9-albino	G	del	G	A
CrA10-albino	G	del	G	A
A. tigrinum 2674	G	del	G	A
A. tigrinum 2675	G	del	G	A
A. tigrinum 2677	G	del	G	A
A. tigrinum 2679	G	del	G	A

thrsp Nucleotide Position

	76	80	115	125	216	238-261
CrA2-wildtype	T	G	A	A	A	ins
CrA6-wildtype	T	G	A	A	A	ins
CrA9-albino	C	C	G	C	G	del
CrA10-albino	C	C	G	C	G	del
A. tigrinum 2674	C	C	G	C	G	del
A. tigrinum 2675	C	C	G	C	G	del
A. tigrinum 2677	C	C	G	C	G	del
A. tigrinum 2679	C	C	G	C	G	del

tyr Nucleotide Position

	187	279	298	316	341	376	400	462	523	530	554
CrA2-wildtype	C	G	A	C	C	C	T	G	C	C	T
CrA6-wildtype	C	G	A	C	C	C	T	G	C	C	T
CrA9-albino	G	A	G	A	G	G	A	A	A	T	C
CrA10-albino	G	A	G	A	G	G	A	A	A	T	C
A. tigrinum 2674	G	A	G	A	G	G	A	A	A	T	C
A. tigrinum 2675	G	A	G	A	G	G	A	A	A	T	C
A. tigrinum 2677	G	A	G	A	G	G	A	A	A	T	C
A. tigrinum 2679	G	G/A	A/G	A	G	C/G	T/A	G/A	A	T	C

sytl2 Nucleotide Position

	109	197	209	259	285	348	349	350	382	432	534
CrA2-wildtype	C	A	C	C	C	-	-	G	T	T	G
CrA6-wildtype	C	A	C	C	C	-	-	G	T	T	G
CrA9-albino	T	T	A	T	A	A	T	T	C	G	A
CrA10-albino	T	T	A	T	A	A	T	T	C	G	A
A. tigrinum 2674	T	T	A	T	A	A	T	T	C	G	A
A. tigrinum 2675	T	T	A	T	A	A	T	T	C	T/G	A
A. tigrinum 2677	T	T	A	T	A	A	T	T	C	T/G	A
A. tigrinum 2679	T	T	A	T	A	A	T	T	C	T/G	A

Supplementary Figure S3. Single nucleotide polymorphisms identified for *nox4, thrsp, tyr,* and *styl2* using two axolotl wild type and two albino sibs, and four *A. tigrinum* larvae captured from a pond in Minnesota.

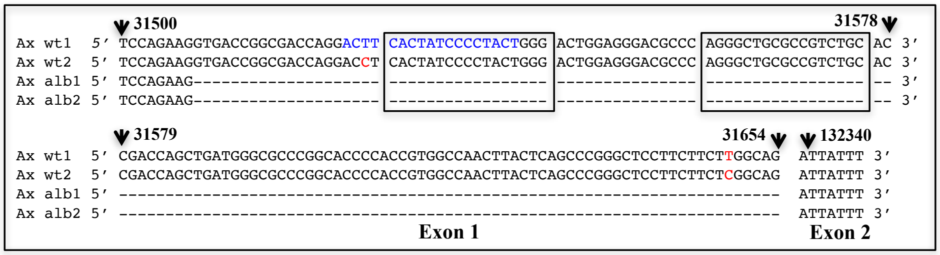

Supplementary Figure S4. Albino is associated with a deletion that lacks 142 bp of tyr exon 1. Fei et al36 targeted this region with a TALEN (boxed regions correspond to left and right TALEN arm-binding sites) and a CRISPR-Cas9 gRNA (blue nucleotides) to create mosaic axolotl albinos. The nucleotide positions are from GenBank Locus KU684456.

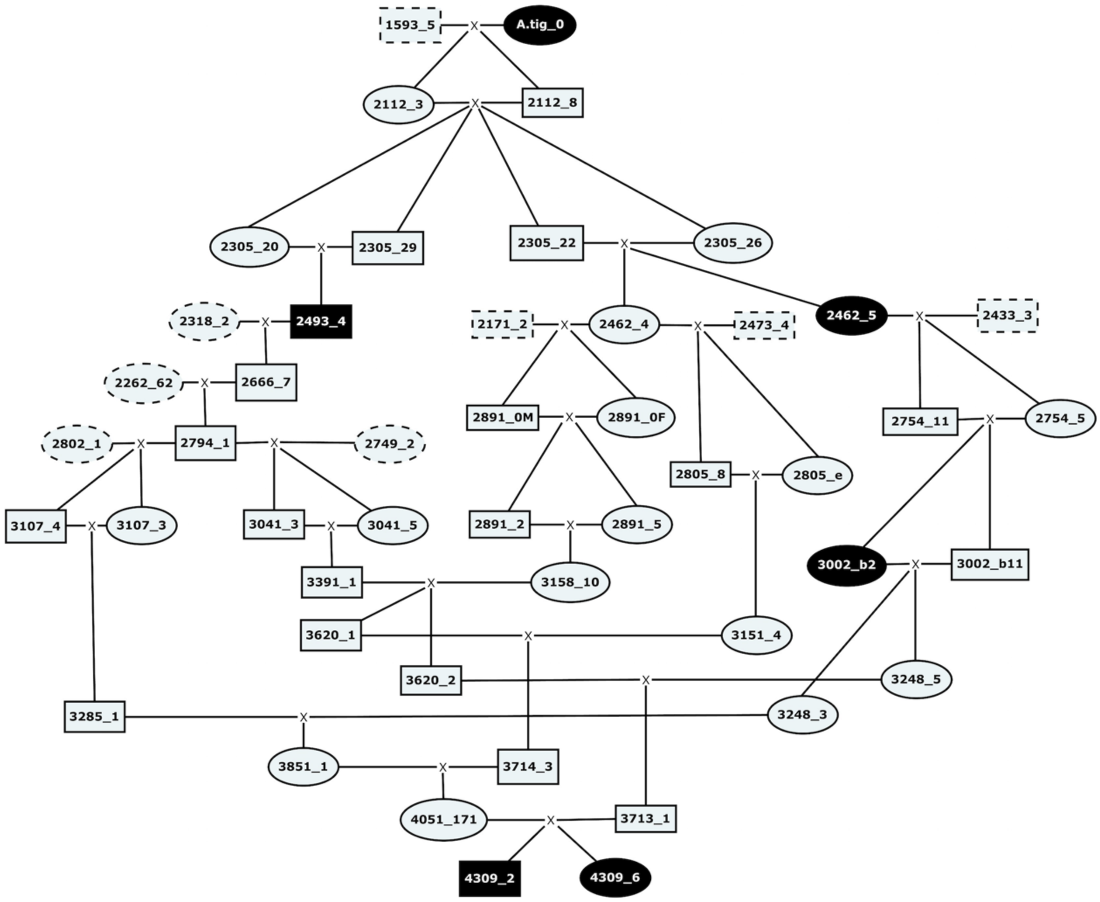

Supplementary Figure S5. Three lineages deriving from the initial *A. mexicanum* x *A. tigrinum* cross that established albino in the AGSC. The albino tiger salamander is identified as A.tig_0. All other individuals have identifiers denoting spawn (prefix) and individual (suffix) numbers. Males are illustrated with squares while females appear as circles. A dotted square or circle indicates an axolotl unrelated to the albino Tiger salamander. Matings are indicated with an “X”. Uncolored circles and squares indicate wild-type phenotype and dark colored circles and squares indicate albino phenotype. Two F1 hybrids were mated to generate male and two female offspring that were crossed to produce F3 offspring. An F3 albino male (2493_4) was backcrossed (broken circle) to an axolotl and backcrosses were performed for 2 successive generations, with two different backcrosses performed in the 3rd generation. In theory, the amount of tiger salamander DNA in this lineage (Founder Lineage 1; FL1) was reduced to 1/32. Two F3 females from the other F2 cross were reared to maturity, one albino (2462_5) and one wild type (2462_4) that carried the albino allele. The albino female was backcrossed to two different axolotls and the other crossed to still another different axolotl (Founder Lineages 2-4: FL2-4). However, unlike FL1, FL2-4 descendants were not backcrossed further, and thus in theory, tiger salamander DNA was only reduced to 25% in these lineages. Individuals from FL2-4 were subsequently crossed into FL1 (and vice versa), thus possibly increasing the representation of tiger salamander DNA in individuals (e.g. 4309_2; 4309_6) that generated the initial albino breeders.

Supplementary Table S6. Thirty-five genes located between *nox4* and *thrsp* on human chromosome 11. The relative order of these genes is likely conserved in axolotl.

**Start BP**	**Stop BP**	**Gene Symbol**
78063861	78068357	THRSP
78068347	78080219	NDUFC2
78100942	78139660	ALG8
78171243	78188817	KCTD21
78188812	78217644	USP35
78215290	78417822	GAB2
78435961	78574864	NARS2
78653283	79440989	TENM4
82732004	82733864	FAM181B
82824367	82901691	PRCP
82901695	82934659	DDIAS
82973133	83071923	RAB30
83157095	83185794	PCF11
83193739	83256099	ANKRD42
83259093	83286407	CCDC90B
83455012	85628534	DLG2
85628573	85636539	TMEM126B
85647919	85656553	TMEM126A
85657564	85665138	CREBZF
85683844	85686277	CCDC89
85694221	85811159	SYTL2
85855055	85920021	CCDC83
85957171	86069881	PICALM
86244544	86285420	EED
86302211	86345943	C11orf73
86374736	86423109	CCDC81
86441108	86672636	ME3
86791059	86952910	PRSS23
86945675	86955398	FZD4
87037844	87328834	TMEM135
88113247	88175467	RAB38
88293592	88337773	CTSC
88504576	89065961	GRM5
89177858	89295759	**TYR**
89324353	89589611	NOX4

A. PCR primers used to amplify DNA fragments for *nox4, tyr, sytl2,* and *sytl2*.

Primer ID	Primer Sequence	PCR Size	Reference Sequence
NOX4_5.2	CTGCAATATTAGCGACAACTCTTTTA		Sal-Site
NOX4_3.2	CTGTTACACTAGAAAAGCAACGAAAA	717 bp	V4contig158118
TYR_5.2	CTTACTCCTGGAAGCAGAAGACTATC		GenBank
TYR_3.6	GTTGCTAGTTAGTTCCCCTTGAATC	720 bp	Locus KU684456
SYTL2_5.1	ACCTCCTATGAAGCTTCCCTGT		Sal-Site
SYTL2_3.3	GACAGTGGCTCTGCTTAAAATCAT	647 bp	V3contig63136
THRSP_5.1	CTATGTACTTAACCGCTTGACCAAC		Sal-Site
THRSP_3.1	GGAACACAGAATACAGGATAACAGAC	319 bp	V3Tig_NM_003251_Contig_1

B. PCR Primers used for *tyr* 5' RACE.

Primer ID	Primer Sequence	Reference Sequence
OCA1_GSP3.2	CAATCCAAGTACAGGAATAGCAAAC	GenBank
OCA1_GSP3.3	ACTTATAGACCGGTTTGCTATTCCT	Locus KU684456

C. PCR Primers used to amplify *edn3* cDNA.

Primer ID	Primer Sequence	PCR Size	Reference Sequence
EDN3_CDS_5.2	GGATTGTGTCACCCAGGATTG	591 bp – *edn3*	GenBank
EDN3_CDS_3.1	GTCCCTCTCTTGATGGTCTATTTGT	451 bp – *edn3*[d]	Submitted

D. Real-Time PCR Primers used to amplify *edn3* and *polr2l* transcripts.

Primer ID	Primer Sequence	PCR Size	Reference Sequence
Edn3_QRT_5.2	AATGCGTCTACTACTGCCACCT		GenBank
Edn3_CDS_3.2	GCCATCTTGTAAGGAATCAAAGTCTC	520 bp	Submitted
POLR2L_5.1	TCTGATCGAGAAACTTTTGAATTATG		Sal-Site
POLR2L_3.1	TCTTTGCAGATAAAATGAAAGTTGAC	339 bp	V4 contig253351

E. Universal M13 Primers used to sequence clones.

Primer ID	Primer Sequence	Reference Sequence
M13F	GTAAAACGACGGCCAGT	pGEM
M13R	CAGGAAACAGCTATGAC	pGEM

Supplementary Figure S8. List of primers used for PCR, cDNA synthesis, 5' RACE, and DNA sequencing.

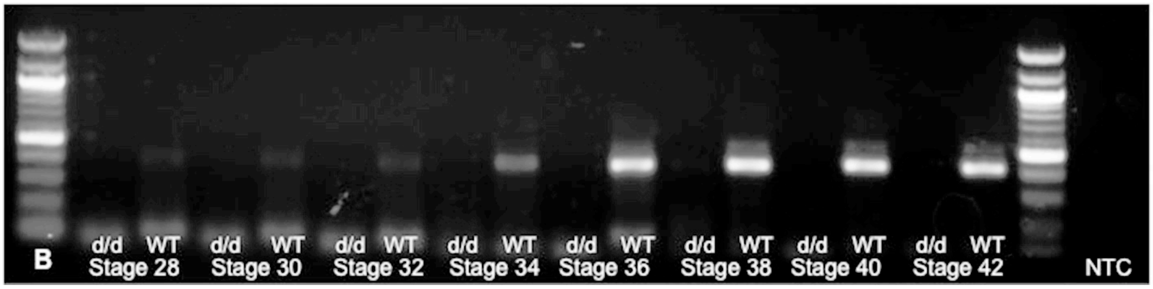

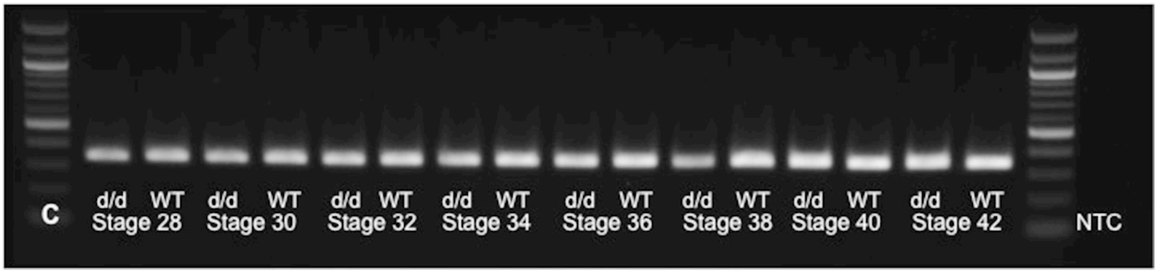

Supplementary Figure 9. Uncropped gel images corresponding to Figure 1C. Upper, *edn3*. Lower, *RNApol2*. NTC, no template control in right-most lanes.
